# Swimming under elevated hydrostatic pressure increases glycolytic activity in gas gland cells of the European eel

**DOI:** 10.1371/journal.pone.0239627

**Published:** 2020-09-30

**Authors:** Gabriel Schneebauer, Constantin Lindemann, Victoria Drechsel, Lasse Marohn, Klaus Wysujack, Elena Santidrian, Ron Dirks, Reinhold Hanel, Bernd Pelster

**Affiliations:** 1 Institute of Zoology, University of Innsbruck, Innsbruck, Austria; 2 Center for Molecular Biosciences, University Innsbruck, Innsbruck, Austria; 3 Thünen Institute for Fisheries Ecology, Bremerhaven, Germany; 4 Future Genomics Technologies, Leiden, The Netherlands; Karlsruhe Institute of Technology, GERMANY

## Abstract

In spite of many decades of research, the spawning migration of the European eel *Anguilla anguilla* from the European coast to the Sargasso Sea remains a mystery. In particular, the role of the swimbladder as a buoyancy regulating structure is not yet understood. In this study, we exercised silver eels in a swim tunnel under elevated hydrostatic pressure. The transcriptome of gas gland tissue of these exercised eels was then compared to the known transcriptome of not exercised (control) silver eel gas gland cells. Due to the high infection rate of the eel population with the swimbladder parasite *Anguillicola crassus*, the comparison also included an exercised group of silver eels with a heavily damaged swimbladder, and we compared the previously published transcriptome of not exercised silver eels with a highly damaged swimbladder with the exercised group of silver eels with a heavily damaged swimbladder. The comparisons of unexercised (control) silver eels with exercised silver eels with functional swimbladder (EF), as well as with exercised silver eels with damaged swimbladder (ED), both showed a significant elevation in transcripts related to glycolytic enzymes. This could also be observed within the comparison of unexercised silver eels with a highly infected swimbladder with exercised eels with a damaged swimbladder (DED). In contrast to EF, in ED a significant elevation in transcript numbers of mitochondrial NADH dehydrogenase was observed. While in EF the transcriptional changes suggested that acid production and secretion was enhanced, in ED these changes appeared to be related to thickened tissue and thus elevated diffusion distances. The remarkable number of differentially expressed transcripts coding for proteins connected to cAMP-dependent signaling pathways indicated that metabolic control in gas gland cells includes cAMP-dependent pathways. In contrast to ED, in EF significant transcriptional changes could be related to the reconstruction of the extracellular matrix, while in ED tissue repair and inflammation was more pronounced. Surprisingly, in exercised eels hypoxia inducible transcription factor expression was elevated. In EF, a large number of genes related to the circadian clock were transcriptionally modified, which may be connected to the circadian vertical migrations observed during the spawning migration.

## Introduction

The spawning migration of the European eel has attracted attention of scientists for more than a century but still remains a mystery [1–3]. To reach the spawning grounds in the Sargasso Sea, mature silver eels have to migrate distances between 5,000 and 7,000 km, depending on their starting point in continental Europe or Northern Africa. The original hypothesis that the spawning migration, starting in fall, would last about 3.4 to 6 months [4] is questioned in recent studies suggesting that the migration may actually last for more than 12 months [5]. The spawning migration thus is energetically highly demanding since eels do not feed during this journey [4, 6].

Anguilliforms are physostome teleosts, but their ductus pneumaticus is functionally closed, so that in terms of buoyancy regulation eels behave as physoclists, i.e. they fill their swimbladder by diffusion of gas molecules from the swimbladder circulation, and gas is removed from the bladder in a modified ductus pneumaticus serving as a resorbing bladder [7]. Essential for gas secretion is the functioning of so-called gas gland cells in the swimbladder epithelium, which are specialized for the production of acid (lactic acid and CO_2_), even in the presence of hyperbaric oxygen partial pressures, to reduce the oxygen carrying capacity of the hemoglobin (Root effect). This results in an initial increase in gas partial pressures (oxygen and CO_2_) [8, 9]. This initial increase in gas partial pressures is then multiplied in a blood vessel arrangement for countercurrent multiplication, a rete mirabile [7, 10, 11]. In contrast to many other fish [12], in the eel swimbladder rete mirabile and gas gland cells are spatially separated, so that their individual contribution to the generation of high gas partial pressures can be assessed. Therefore, the eel swimbladder has been used as a model system for swimbladder function [7].

Recent tracking studies with migrating European and American eels consistently revealed extensive diurnal vertical migrations covering several hundred meters of depth [5, 13–16]. Due to the changes in hydrostatic pressure, vertical migrations over a depth range of several hundred meters result in significant changes in volume of the flexible walled swimbladder. In order to keep the status of neutral buoyancy, the volume of the swimbladder must be kept constant [17, 18], so that significant changes in gas secretion and absorption are required. Although the swimbladder is filled only by diffusion, stimulation of gas secretion requires energy, glucose as source for acid production, and sufficient amounts of oxygen, because oxygen is the main gas filling the swimbladder [19]. Calculating the amount of glucose that would be required for acidification of the blood and the amount of oxygen that would be required to keep the swimbladder volume constant during daily vertical migrations over a period of several months without feeding, revealed that it is impossible for the European eel to perform these daily depth changes in a status of neutral buoyancy [20]. It was therefore concluded that the eel is in a status of neutral buoyancy in the lower depth range during the night and negatively buoyant during daytime, when travelling at greater depth [20]. This would clearly reduce the role of the swimbladder as a buoyancy structure during the spawning migration and raises the question whether the swimbladder is an important structure during the spawning migration, or whether it might even be dispensable.

On the other hand, it is long known that the rete mirabile, essential for countercurrent multiplication and therefore determining the maximum partial pressure that can be achieved in the swimbladder, increases in size during silvering in eels, i.e. when eels change from yellow eels (the continental feeding stage) to silver eels (the migrating stage) [21–23]. Therefore, maturation of eels from the yellow eel stage, living in continental freshwater, to the silver stage prepared for the spawning migration in the Atlantic Ocean, is assumed to be connected to a significant improvement of swimbladder function [24, 25].

To assess the role of the swimbladder during the spawning migration we swam eels in a swim tunnel under elevated hydrostatic pressure. Swimbladder tissues were sampled immediately following decompression. We compared the gas gland cell transcriptome of these eels with that of silver eels kept under atmospheric pressure in the aquarium tank. A significant fraction of the European eel has a swimbladder infested with the nematode *Anguillicola crassus* [26], which causes severe damage to the swimbladder tissue and impairs the proper function of this organ [27, 28]. Therefore, we additionally included exercised eels with a heavily damaged swimbladder in this study and compared their transcriptome with the transcriptome of silver eels that were not exercised, either with a functional or a heavily infected swimbladder.

## Material and methods

Silver eels were caught by commercial fishermen in the Schlei Fjord, Lake Plön, and in the River Ems in accordance with the fisheries legislation of the Federal States Schleswig-Holstein (Schlei, Lake Plön) and Lower Saxony (River Ems). Fishing was conducted in the frame of the Eel Management Plans for the River Basin Districts "Ems" and "Schlei/Trave" according to the EU "Eel Regulation" (Council Regulation (EC) No. 1100/2007 establishing measures for the recovery of the European eel stock). Fish were kept in an outdoor freshwater basin at the Thünen Institute of Fisheries Ecology in Ahrensburg or Bremerhaven, Germany, for at least four weeks prior to the experiments. According to recent studies [29, 30], the European eel is a panmictic (i.e. random mating) species and therefore the different sampling points should not bias the results of this study. Table 1 shows the morphometrics of the animals chosen for the experiments, with the silvering stages calculated according to the index of Durif et al. [31].

**Table 1:** Morphometrics of exercised silver eels.

Body mass (g)	1174.6 ± 321.2
Body length (cm)	83.9 ± 8.8
Pectoral fin length (mm)	41.1 ± 6.1
Horizontal eye diameter (mm)	11.2 ± 1.3
Vertical eye diameter (mm)	10.6 ± 1.2
Silver index	4.1 ± 0.3

Silver index calculated according to Durif [31]. Overall mean values ± S.D.; *N* = 14.

Eels were also grouped according to the physical integrity of their swimbladders after infection with *Anguillicola crassus*, as classified by Hartmann [32]. Previously heavily infected swimbladders had a thickened, multilayered epithelium with opaque appearance as described by Würtz and Taraschewski [28], contained exudates and very low gas volumes. These swimbladders, classified as Hartmann degree 4 or 5 [32], were defined as damaged. Healthy swimbladders, classified as Hartmann degree 1, were defined as functional. Experiments were performed in compliance with the Austrian law and the German law (permission No.: §7(2) 142) and were approved by the animal experimental committee of the Thünen Institute.

### Swim tunnel experiments

#### Construction of the pressure flow tunnels

The pressure flow tunnels were constructed as three separate, closed pipe systems made of inert and food-safe plastic (PVC-U) with a transparent acrylic glass float chamber in which the water circulation was achieved by an impeller. Each pressure flow tunnel had a length of 4 m. The piping and the swimming chamber of the pressure flow tunnels were designed for simulation of a maximum water depth of 100 m (Ø 20 cm, thickness 2 cm), i.e. a hydrostatic pressure of 11 atm (= 1.1 MPa). Laminar flow was provided by means of a flow rectifier (length 50 cm with 91 holes á Ø 1.3 cm), which was integrated into the filling/extraction opening at the head of the swimming chamber. In order to visualize potential turbulences, the flow profile during the test phase was examined and optimized in a prototype using food color or fluorescein. To avoid problems with the solid blocking effect due to large eels measuring up to 6 cm in diameter (cross-section area: 28.3 cm^2^), the tube diameter was set to 20 cm (cross-section area: 314.2 cm^2^). An electric barrier (24 V with 11.7 KHz pulse width modulation and 100 Hz polarity change for electrolysis avoidance), activated by a centrally positioned light barrier, prevented the fish from resting in the rear part of the acrylic glass swim tunnel. A T-piece, which was connected upstream to the swimming chamber, served as a filling/removal opening. By means of screening grids, which could be moved flexibly to magnets, test animals could easily be removed from the swim tunnel.

To enable long-term studies, all three hyperbaric floating tunnels were connected to a central non-pressurized tank for water treatment with appropriate mechanical and bio-filter systems for microbial degradation of excreted metabolic end products, including control of water NH_3_ and NH_4_^+^ content, oxygenation of the water, regulation of water temperature, and microbial reduction by UV treatment. Water volume of the central tank, including piping and treatment basin, was 1,200 l, while the complete circuit with all swim tunnels held 1,815 l (205 l per tunnel). When the valves were open, the water flowing out of the tunnels circulated through the central tank, where it was treated and then returned to the swim tunnels with pressure pumps. The water volume of a single swim tunnel could thus be exchanged within 1–3 hours, depending on how many tunnels were flushed at the same time. The pressure pumps could build a pressure of up to 1.1 MPa in the swim tunnels, which corresponds to a hydrostatic pressure at 100 m water depth.

A camera and lighting LEDs (daylight and infrared LEDs) were mounted above the central part of the tunnel (length 1.4 m). The daylight lighting consisted of a programmable RGB LED strip (60 LEDs per meter, 2 stripes of 1.5 m length per tunnel, APA102 LED type), so the spectral color and brightness could be controlled depending on daytime and pressure. The light regime was L/D = 14/10 h. Oxygen was measured automatically with the Oxygen Optode 4835 (Aanderaa Data Instruments AS, Bergen, Norway), avoiding PO_2_ values below 80% oxygen saturation. Water temperature and velocity were recorded using appropriate sensors, and water velocity was adjusted depending on the length of the fish in order to achieve a swimming speed of 0.5 bl s^-1^ (body lengths per second). Data from the video recordings as well as all sensor data were stored on a PC using custom prepared software. Via feedback loops, temperature, water velocity, and oxygen saturation of the water were kept constant throughout the experiment.

#### Experimental protocol

Two weeks prior to the experiments, the salinity of the basins was increased at a rate of 7 PSU d^-1^ until reaching 35 PSU. Eels were transferred to the swim tunnel and acclimatized to the new environment at a pressure of 1 bar and a flow rate of 0.1 m s^-1^ for 2 h at a temperature of 14°C. Over a period of 16 h, eels were acclimatized to higher flow rates of up to 0.5 bl s^-1^, which corresponds to the estimated swimming speed of the spawning migration [33]. Pressure was elevated at a rate of 1 bar min^-1^ which corresponds to the natural diving speed of 0.15 m s^-1^ [34] to finally 8 bar. The 24 h experiment was terminated by 8 h of exercise at a velocity of 0.5 bl s^-1^ and a pressure of 8 bar (= 0.8 MPa). At the end of the experiment, pressure was reduced to atmospheric pressure at a rate of 1 bar min^-1^ and water velocity was reduced to 0.1 m s^-1^. Eels were removed from the tunnel, immediately anesthetized with 2-phenoxyethanol (1 ml l^-1^), and subsequently decerebrated and spinally pithed. The swimbladder was dissected and the epithelium, consisting of gas gland cells, was carefully freed from connective tissue. The epithelium was transferred into RNAlater™ solution (Invitrogen by Thermo Fisher Scientific Inc., Waltham, USA) and stored at -80°C until further use.

#### RNA isolation and Illumina RNAseq analysis

Total RNA was isolated using the Qiagen miRNeasy Mini kit and a TissueRuptor according to the manufacturer’s instructions (Qiagen, Venlo, Netherlands). Integrity and quality of the RNA were checked on an Agilent Bioanalyzer 2100 total RNA Nano series II chip (Agilent, Amstelveen, Netherlands). Illumina RNAseq libraries were prepared from 0.5 μg total RNA using the Illumina TruSeq Stranded mRNA Library Prep according to the manufacturer’s instructions (Illumina Inc. San Diego, CA, USA). All RNAseq libraries (150–750 bp inserts) were sequenced using an Illumina NovaSeq6000 system as 2 × 150 nucleotides paired-end reads or an Illumina HiSeq2500 system as 1 × 50 nucleotide single-reads according to the manufacturer’s protocol. Image analysis and base calling were done using the Illumina pipeline.

#### Illumina data processing

Data processing was performed as described previously [35, 36]. Briefly, reads (15 million per sample) were aligned to the draft genome sequence of European eel [37] using TopHat (version 2.0.13) [38]. Secondary alignments of reads were excluded by filtering the files using SAMtools (version 1.2 using htslib 1.2.1) [39]. Aligned fragments per predicted gene (also referred to as transcripts) were counted from SAM alignment files using the Python package HTSeq (version 0.6.1p1) [40]. To make comparisons across samples possible, fragment counts were corrected for the total amount of sequencing performed for each sample. As a correction scaling factor, library size estimates, determined using the R/Bioconductor (release 3.3.2) package DESeq [41], were employed. Read counts were normalized by dividing the raw counts obtained from HTSeq by its scale factor. The term “relative expression value” refers to these normalized read counts.

Differentially expressed genes between silver eels (unexercised controls, SC) and silver eels with a functional or a damaged swimbladder exercised in the swim tunnel were identified using DESeq, the cut-off for significance was set to p < 0.05. The comparison of unexercised (control) silver eels, with exercised silver eels with functional swimbladder is defined as EF, and with exercised silver eels with damaged swimbladder is defined as ED, respectively. Similarly, the comparison of the transcriptome of unexercised silver eels with damaged swimbladder (SD) with the transcriptome of exercised silver eels with a damaged swimbladder is defined as DED. Data for unexercised silver eels (SC, SD) were taken from a previous study [42].

Gene ontology (GO) enrichment analysis was performed using Database for Annotation, Visualization and Integrated Discovery (DAVID) software tools (version 6.7; https://david.ncifcrf.gov) [43]. An EASE score of 0.05 along with standard default settings was used, and the resulting categories were considered significant at p < 0.01. The Reactome database (https://reactome.org) [44] was used for classification of genes into broad pathways. For a detailed pathway and biological process analysis of differentially expressed genes, GO *biological process* and *molecular function* were searched for genes related to a specific metabolic pathway (like, for example, energy metabolism, membrane transport, apoptosis), and the GO *cellular component* was searched for genes related to a specific cellular structure or organelle (like, for example, extracellular matrix or mitochondrial).

## Results

All eels swam in the swim tunnel for 24 hours with a water velocity of 0.1–0.5 bl s^-1^ during the first 16 h. During the last 8 h eels had to swim at a speed of 0.5 bl s^-1^ and a hydrostatic pressure of 8 atm. There was no obvious difference in the performance of silver eels with a functional swimbladder and eels with a damaged swimbladder.

A total of 14 cDNA libraries were constructed from swimbladder RNA of 6 exercised silver eels with a functional swimbladder and 8 exercised silver eels with a heavily damaged swimbladder. The damaged swimbladders had a multilayered epithelium (Hartmann 4 & 5) and contained only minimal traces of gas or no gas at all. On average, a cDNA library was sequenced at a depth of 23,805,782 raw reads. Alignment to the European eel reference genome [37] resulted in about 61% mapped genes (14,568,232 reads). These transcriptomes were compared with the transcriptome of 5 control silver eels not exercised in the swim tunnel (SC), obtained from a previous study comparing yellow and silver eels [42]. In addition, the previously published transcriptome of unexercised silver eels with a heavily damaged swimbladder [42] was also compared with the transcriptome of exercised silver eels with a heavily damaged swimbladder (Hartmann degree 4 or 5), the results of this comparison are presented as DED.

The comparison of the transcriptome of non-exercised uninfected silver eels (SC) with the transcriptome of exercised eels with functional swimbladder at the significance level of p < 0.05 revealed 3,476 differentially expressed genes (DEGs), out of which 1,542 (= 44%) were down-regulated (S1 Table). Comparing the transcriptome of SC with the transcriptome of exercised eels with damaged swimbladder at the significance level of p < 0.05 revealed 3,724 DEGs, out of which 1,253 (34%), were down-regulated (S2 Table). Comparison of non-exercised infected silver eels (SD) against exercised eels with damaged swimbladder revealed 8,197 DEGs, 4,161 of these DEGs were elevated, and 4,036 DEGs were reduced (S3 Table).

**Fig 1:**
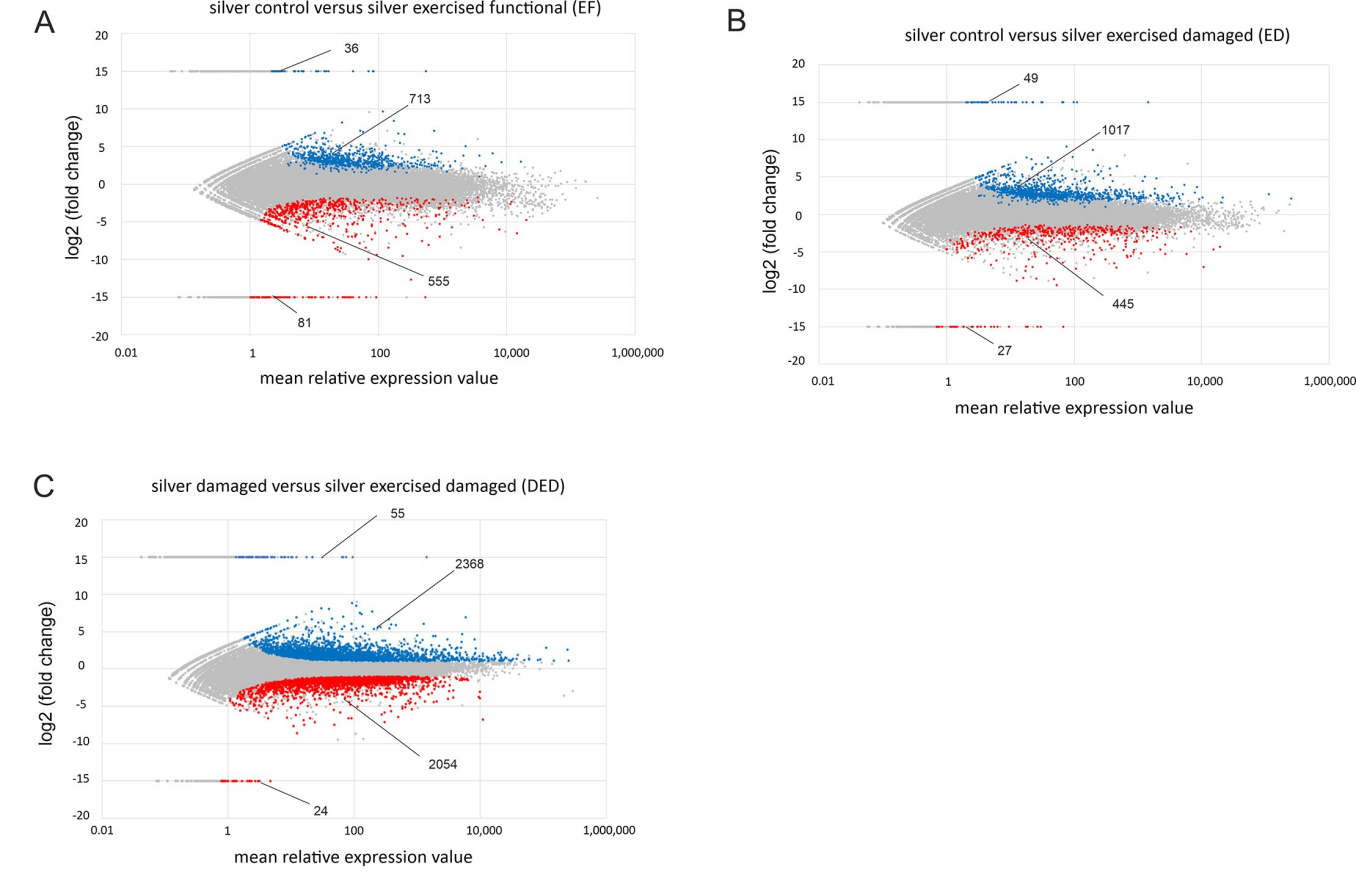
MA-plot of DEGs. (A) MA-plot of DEGs from control swimbladder tissue of unexercised silver eels compared with exercised silver eels, (B) from control silver eel swimbladder tissues and exercised silver eels with damaged swimbladder tissue and (C) from unexercised silver eels with damaged swimbladder compared with exercised eels with damaged swimbladder. Blue and red dots denote up- and down-regulated genes, respectively (pval < 0.01). Grey dots denote non-regulated genes (pval > 0.01). Newly activated or newly deactivated genes were arbitrarily assigned to a log2 (fold change) = 15 or -15, respectively. The value of log2 (fold change) for all genes was analyzed as log2 (exercised/unexercised). Numbers indicate the numbers of DEGs either elevated or reduced in transcription.

Plotting the log2-fold change of in exercised eels DEGs versus the mean expression values of the genes (MA-plot; Fig 1) revealed the differing numbers and patterns of up- and down-regulated, and newly activated and deactivated genes when comparing control eels with a functional swimbladder (SC) against exercised eels with a functional swimbladder (Fig 1A), or against exercised eels with a damaged swimbladder (Fig 1B). For clarity, the level of significance was reduced to p < 0.01 for this analysis. Although the total number of DEGs was similar in EF (1,385 DEGs) and ED (1,538 DEGs), their distribution was rather different. In EF, the number of up-regulated or activated genes (749 DEGs) was close to the number of down-regulated or deactivated genes (636 DEGs), whereas in ED 1,066 genes were up-regulated or activated, and 472 DEGs were down-regulated or deactivated. Comparing SD with exercised eels with damaged swimbladder revealed a much higher number of affected genes (4,501 DEGs), with 2,423 up-regulated and 2,078 down-regulated genes (Fig 1C).

**Fig 2:**
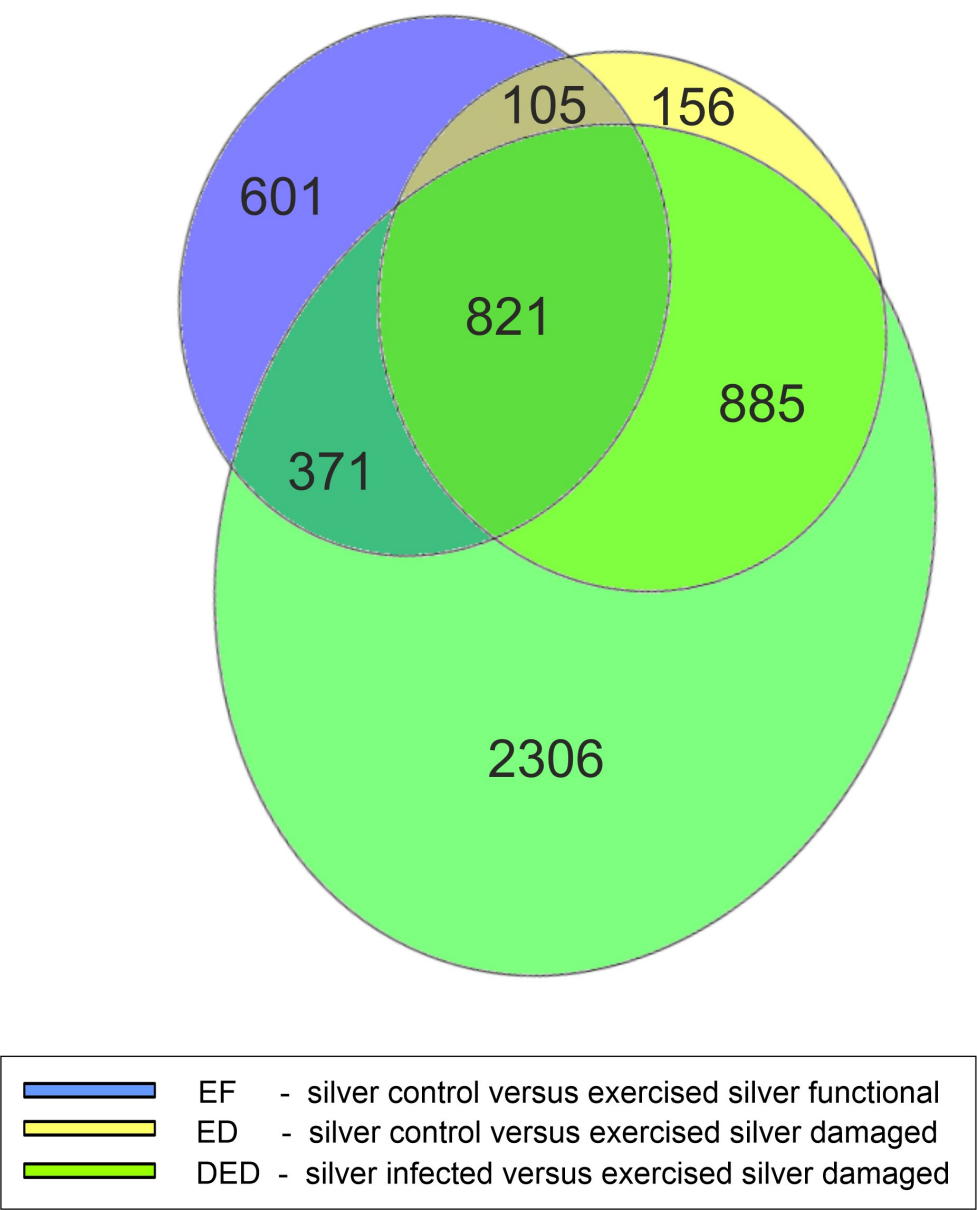
Number and overlap of differentially expressed genes. Venn diagram showing the total number of annotated genes of the comparisons (p < 0.05) between silver control (SC) with exercised eels with functional swimbladder in blue, SC with exercised eels with damaged swimbladder in yellow, and silver control eels with damaged swimbladder (SD) with exercised eels with damaged swimbladder in green, with the corresponding overlaps. Diagram was generated with euler*APE* (http://www.eulerdiagrams.org/eulerAPE/) [45].

A Venn diagram revealed that 49% of the annotated DEGs detected in EF were also significantly modified in ED (Fig 2). 32% of DEGs (601 DEGs) detected in EF were not modified in eels with a damaged swimbladder. 261 of the in ED identified DEGs were not differentially expressed when the transcript of exercised fish was compared with unexercised silver eels with damaged swimbladder. The diagram clearly showed that most of the DEGs identified were connected to damage of the swimbladder.

**Fig 3:**
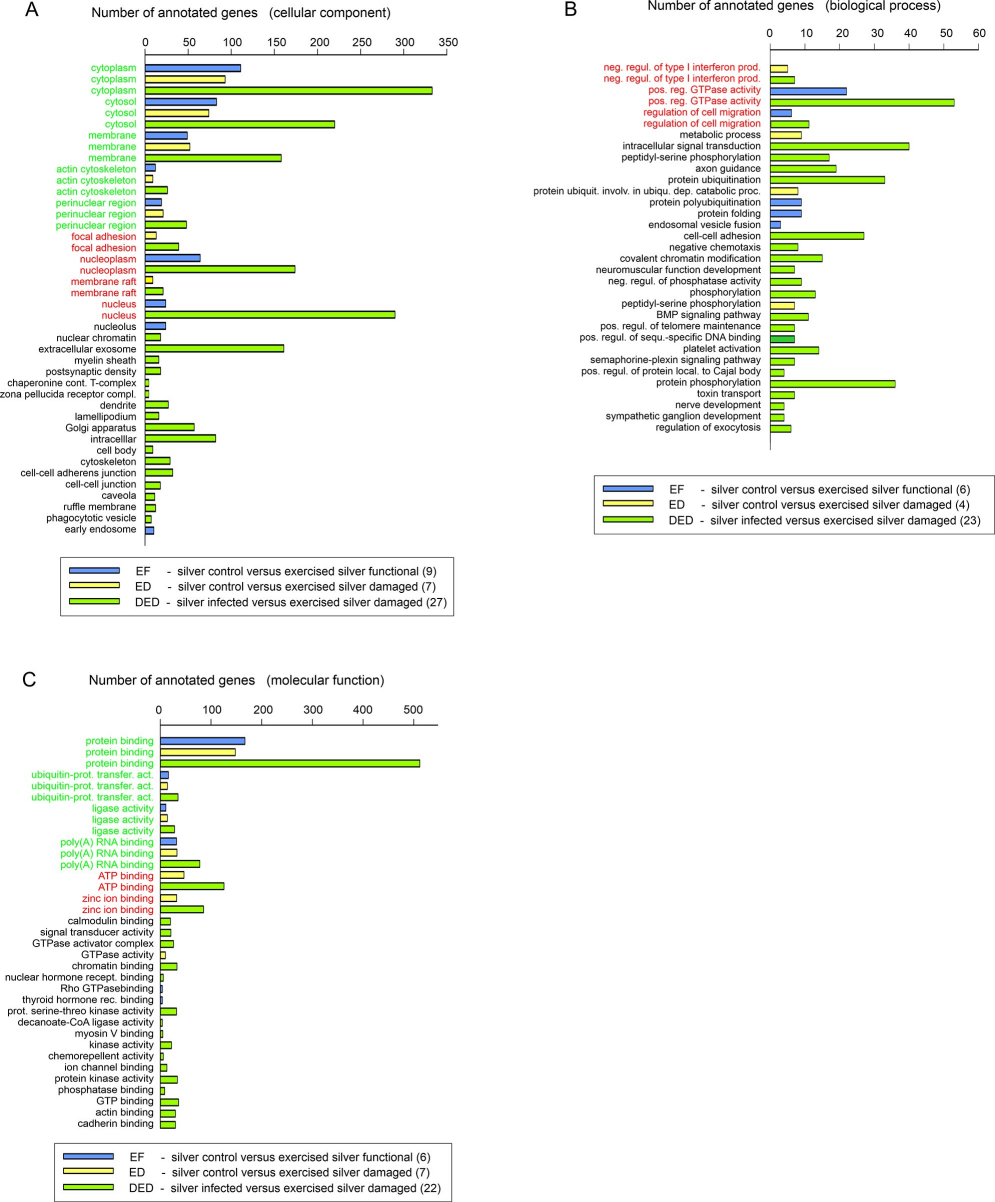
GO enrichment analysis. (A) Significantly affected *cellular components* identified by GO enrichment analysis of DEGs at the level of p < 0.01 for the comparison of control silver eels (SC) versus exercised silver eels with a functional swimbladder (blue) (EF) and the comparison of silver control eels (SC) versus exercised silver eels with a damaged swimbladder (yellow) (ED). The comparison of infected unexercised silver eels (SD) with exercised eels with a damaged swimbladder is shown in green (DED). *Cellular components* affected in two groups are listed in red, components affected in all three groups are listed in green. (B) Significantly affected *biological processes* identified by GO enrichment analysis of DEGs at the level of p < 0.01, EF values are shown in blue, ED values in yellow, and DED in green. *Biological processes* affected in two groups are listed in red, processes affected in all three groups are listed in green. (C) Significantly affected *molecular functions* identified by GO enrichment analysis of DEGs at the level of p < 0.01, EF values are shown in blue, ED values in yellow, and DED in green. *Molecular functions* affected in two groups are listed in red, functions affected in all three groups are listed in green.

We used DAVID for GO enrichment analyses to detect DEGs in exercised eels compared to SC eels at the level of p < 0.01. In EF, 734 DEGs were annotated, out of which 708 genes were assigned with a unique *cellular component*, 676 genes with a unique *biological process*, and 656 genes with a unique *molecular function*. In ED, 771 DEGs were annotated, out of which 747 genes were assigned with a unique *cellular component*, 693 genes with a unique *biological process*, and 679 genes with a unique *molecular function*. In DED, 2,529 annotated DEGs were identified, out of which 2,406 genes were assigned with a unique *cellular component*, 2,292 genes with a unique *biological process*, and 2,208 genes with a unique *molecular function*. In EF, 9 different *cellular components* were significantly (p < 0.01) affected, 5 of which were also affected in ED (Fig 3A). In DED, 27 *cellular components* were significantly affected. 6 *biological processes* were significantly modified in EF, but there was no overlap with the 4 *biological processes* significantly affected in ED (Fig 3B). Again, with 23 a much larger number of *biological processes* were significantly affected in DED. Looking at the GO domain *molecular function*, protein binding, ubiquitin-protein binding, ligase activity and poly(A) RNA binding were affected in both EF and ED. 22 *molecular functions* were significantly affected in DED (Fig 3C).

Pathway analysis using the Reactome pathway browser at the level of p < 0.01 focusing in particular on metabolic pathways, signal transduction, extracellular matrix, and also on cell death and immune response, revealed that about 10–33% of the genes expressed in the various pathways in SC were differentially expressed in either EF or ED (Table 2). Obvious differences between eels with a functional and a damaged swimbladder were detected with respect to the extracellular matrix, where 24% of the genes were differentially expressed in EF, but only 18% in ED. On the other hand, in ED all pathways related to cell damage, cell death or cell repair showed a higher percentage of DEGs. Genes related to organelle biogenesis and maintenance, however, showed with 18% in EF compared to 10% in ED a much higher percentage of DEGs in EF. Noteworthy was the high level of DEGs in genes related to the circadian clock, namely 33% in EF and 25% in ED (Table 2). As to be expected based on the higher number of DEGs identified in DED, for all pathways a higher number of affected genes was detected in DED. For most pathways the number of DEGs in DED was about twice as high compared to ED. For disease-related DEGs, however, in DED the number was almost three times higher than in ED (Table 2).

**Table 2:** Total number of genes represented in a defined pathway in the Reactome pathway database, total number of genes within a defined pathway expressed in control silver eels (SC), and the total number of in exercised eels differentially expressed genes (EF, ED, DED).

	Total number in pathway	Total number expressed in SC	Diff. expressed in EF		Diff. expressed in ED		Diff. expressed in DED	
Metabolism	3,636	901	139	(15%)	148	(16%)	334	(37%)
Metabolism of proteins	2,354	955	170	(18%)	183	(19%)	331	(33%)
Metabolism of RNA	782	369	58	(16%)	61	(17%)	118	(32%)
Signal transduction	3,303	1,342	220	(16%)	241	(18%)	517	(39%)
Extracellular matrix	329	120	29	(24%)	21	(18%)	49	(40%)
Circadian clock	104	65	21	(33%)	16	(25%)	27	(43%)
Organelle biogenesis and maintenance	335	172	31	(18%)	17	(10%)	58	(34%)
Programmed cell death	197	90	20	(22%)	22	(24%)	39	(43%)
Disease	1,552	539	87	(16%)	101	(19%)	311	(58%)
Immune system	2,822	1,063	192	(18%)	221	(21%)	457	(43%)
DNA repair	367	179	26	(15%)	28	(16%)	52	(29%)
DNA replication	141	71	7	(10%)	12	(17%)	24	(34%)

Within each pathway, the number of differentially expressed genes (p < 0.01) is listed for exercised silver eels with functional swimbladder as compared with control eels (EF), and for exercised silver eels with damaged swimbladder as compared with control eels (ED). The comparison of exercised silver eels with damaged swimbladder (DED) with unexercised silver eels with a highly infected swimblader (SD) is also included. The proportion of differentially expressed genes of the total number of expressed genes in silver eels in the respective pathway is given in brackets (% value). Reactome database (https://reactome.org) [44].

### Metabolic processes

In a more detailed analysis, at the level of p < 0.05, of all 3,476 in EF identified DEGs, 422 genes (representing 12.1%) were assigned with the GO *biological process* ‘metabolic process’ and 64% of them were upregulated. 43 DEGs were related to ‘energy metabolism’ and 60% of them were upregulated. A large number of genes connected to glucose and energy metabolism were significantly elevated, like glucose-bisphosphate synthase (*pgm2l*), phosphoglucomutase (*pgm*), 6-phosphofructo-2-kinase (*f263*), glycerol-3-phosphate dehydrogenase (*gpda*), pyruvate kinase (*kpyk*), and lactate dehydrogenase (*ldha*) (Table 3 shows a selection of DEGs assigned to the term ‘metabolic process’). In SC, a relative expression value of 470 was detected for *ldha*, while after exercise under elevated hydrostatic pressure the relative expression value increased to 6,480, representing a 13.8-fold increase. Several genes related to glycogen metabolism like glycogen phosphorylase (*pygl*, *pygm*) also showed several-fold elevated relative expression values in EF. In turn, the relative expression values for mitochondrial NADH dehydrogenase (*ndus1* and *nduv2*) were reduced. Carbonic anhydrase (*cahz*) was highly expressed and the relative expression value increased from 1,309 to 5,908 reads in EF (Table 3).

**Table 3:** Selection of DEGs in EF, ED, and DED assigned to the term ‘metabolic process’, and the relative expression value of the respective genes detected in control groups SC and SD.

Metabolic processes	SC	EF	ED	SD	DED
	relative expression value	fold change	fold change	relative expression value	fold change
**Glucose-Bisphosphat Synthase**					
*pgm2l (2x)*	105–1655	↑ (1/2)	↑	100–925	↑
**Phosphoglucomutase**					
*pgm (3x)*	5	↑ (1/3)	↑ (1/3)	10–965	↑
**6-Phosphofructo-2-Kinase**					
*f263*	700	↑	-	-	-
**Glycerol-3-Phosphate Dehydrogenase**					
*gpda (3x)*	185–3000	↑ (1/3)	↑	255–4105	↑
**Pyruvate Kinase**					
*kpyk (5x)*	10–800	↑ (2/5)	↑ (4/5)	120–4770	↑
**Lactate Dehydrogenase**					
*ldha (4x)*	470–860	↑↑ (1/4)	↑ (2/4) - ↑↑ (1/4)	750–30730	↑
**Glycogen Phosphorylase**					
*pygl (2x)*	5–115	↑ (1/2) - ↑↑ (1/2)	↑↑ (1/2)	5	↑ (1/2)
*pygm (3x)*	5–20	↑↑ (1/3)	↓ (2/3)	5–90	↓
**Mitochondrial NADH Dehydrogenase**					
*ndus2 (3x)*, *ndus3 (3x)*, *ndus4*, *nduv1 (2x)*	50–355	↓ (2/9)	↑ (3/9)	15–180	↑
**Carbonic Anhydrase**					
*cahz*, *cah9*	1160–1310	↑ (1/2)	↑ (1/2)	1055–13215	↑
*cah12*	90	-	↓	60	↓
**Hexokinase**					
*hxk1 (2x)*, *hxk4*	5–465	-	↓ (2/3)	5–13020	↑ (1/3) - ↓ (2/3)
*hxk2 (3x)*	< 5–40	-∞	↑	15–70	↑

Arrows indicate increased (upwards) or decreased (downwards) expression values; a single arrow indicates up to 10-fold elevation/reduction, two arrows indicate more than 10-fold elevation/reduction. Numbers in brackets give the number of transcripts for the respective gene. If two numbers are listed, the first number represents the number of significantly affected transcripts.

Within ED 3,724 genes were differentially expressed and 1,253 (= 33%) of these genes were down-regulated. The percentage of genes involved in ‘metabolic processes’ was even higher (13.9%) as compared with EF. Focusing on genes related to ‘energy metabolism’, significant differences were detected as compared with the changes observed in EF. 48 of a total of 56 ‘energy metabolism’ related genes were elevated in ED. In contrast to EF, hexokinase-1 (*hxk1*) was reduced in ED, but the relative expression values of 3 isoforms of hexokinase-2 (*hxk2*) were about 5-fold elevated as compared with silver control eels. Similar to EF, the relative expression values of the glycolytic enzymes *pgm2l*, *kpyk*, and *gpda* were elevated, and lactate dehydrogenase DEGs were also 7.4 and 12.8-fold elevated. Also, mitochondrial genes *ndus2*, *ndus4*, and *nduv1* were elevated, which was not observed in EF. Carbonic anhydrase (*cahz*) was highly expressed and increased from 1,158 to 7,451 corrected reads in ED, while *cah12* was reduced in expression (Table 3). Similar to ED, in DED elevated transcript levels for several glycolytic enzymes were detected, but *ldha* and *pygl* were less affected compared with ED (Table 3). Remarkable was the elevation of *ndus* and *nduv* transcripts in DED, which even exceeded the elevation detected in ED, and which was reduced in EF. Similar to ED, *hxk2* was elevated in DED.

### Membrane transport

To assess DEGs coding for proteins associated with membrane transport, the description of DEGs was searched for the terms ‘ion transport’, ‘membrane transport’, ‘channel’, and ‘ATPase’, whereby the term ‘ATPase’ was restricted to membrane bound ATPases. Table 4 shows a selection of the DEGs identified this way. Among transcripts coding for membrane proteins transporting glucose or lactate, DEGs of *gtr5*, *9*, and *12* as well as of monocarboxylate transporter 1 (*mot1*) were reduced in EF, while DEGs of *gtr1* were elevated in EF and in ED. The by far highest relative expression values for the genes were detected for *gtr1* and *gtr5*. Monocarboxylate transporter 13 (*mot13*) was elevated in expression in ED, while *mot8* was reduced.

**Table 4:** Selection of DEGs in EF, ED, and DED assigned to the term ‘membrane transport’, and the relative expression value of the respective genes detected in control groups SC and SD.

Membrane transport	SC	EF	ED	SD	DED
	relative expression value	fold change	fold change	relative expression value	fold change
**Membrane proteins transporting glucose or lactate**					
*gtr1 (4x)*	5–240	↑ (1/4) - ↑↑ (1/4)	↓↓ (1/4) - ↑ (1/4) - ↑↑ (2/4)	5–260	↑ (1/4) - ↑↑ (2/4) - ↓ (1/4)
*gtr5*	4105	↓↓	-	30	↑
*gtr8*, *gtr11*	-	-	-	40–590	↑ (1/2) - ↓ (1/2)
*gtr9 (2x)*, *gtr12 (2x)*	< 5–15	↑ (1/4) - ↓ (2/4)	-	5–30	↑ (2/4) - ↓ (2/4)
**Monocarboxylate Transporter**					
*mot1*, *mot6*, *mot8*, *mot13*	5–100	↓ (1/4) - ↓↓ (1/4)	↑ (1/4) - ↓ (1/4)	125–385	↑ (1/4) - ↓ (2/4)
*mot2*, *mot7 (2x)*, *mot9*	-	-	-	90–330	↓
**Na+/K+-ATPase subunit α**					
*at1a (7x)*	0–100	↑↑ (1/7) - ∞ (2/7)	↑ (3/7) - ↑↑ (1/7) - ∞ (2/7)	20–5880	↑ (4/7) - ↑↑ (1/7) - ↓ (2/7)
**Na+/K+-ATPase subunit β**					
*nkai*	< 5	↑	↑	< 5	↑
**Ca2+ ATPase**					
*at2b (5x)*	< 5–15	↑ (2/5) - ↑↑ (1/5)	↑ (2/5) - ↑↑ (3/5)	5–80	↑ (3/5) - ↑↑ (1/5)
**V-type ATPase**					
*vatb*, *vat2b*	20–270	↓ (1/2)	↓	30–280	↓ (1/2) - ↓↓ (1/2)
*vatl (2x)*	50	↓ (1/2)	↑ (1/2)	40–90	↑
**Zinc transporter**					
*s39a4*. *s39a5*, *s39a6*, *s39aa*, *s39ad*	< 5–765	↑ (2/5) - ↓ (1/5)	↑ (1/5)	10–1035	↑ (3/5) - ↓ (1/5)
*znt1 (2x)*, *znt2*, *znt6*	5	↓ (1/4)	↑ (2/4) - ↓↓ (1/4)	5–115	↑ (2/4) - ↓ (1/4)
**Purinoreceptor**					
*p2rx1*, *p2rx3*, *p2rx4*	< 5–615	↑ (1/3)	↓ (1/3)	120–615	↑ (1/3) - ↓ (1/3)
*p2rx5*	400	↑	↑	155	↑↑
**Chloride intracellular channel protein**					
*clic2*	-	-	-	1385	↓
*clic4 (2x)*	5–10	↑↑	↑	10–20	↑
**Sodium-hydrogen exchangers**					
*sl9a5*, *sl9a7*, *sl9a8*	> 800	-	-	175	↑ (1/3)
**Voltage-dependent anion-selective channel protein**					
*vdac2 (3x)*, *vdac3 (2x)*	< 5–990	-	↑ (1/5) - ↑↑ (1/5)	5–15420	↑ (4/5) - ↑↑ (1/5)
**Ca2+ channel**					
*ca2d (3x)*	10–30	-	↓ (2/3)	10–150	↓
**Sodium Channel**					
*scn8a*, *scn1b*, *scn4b*	< 5–30	-	↓ (1/3)—-∞ (1/3)	95–170	↓ (2/3)

Arrows indicate increased (upwards) or decreased (downwards) expression values; a single arrow indicates up to 10-fold elevation/reduction, two arrows indicate more than 10-fold elevation/reduction. Numbers in brackets give the number of transcripts for the respective gene. If two numbers are listed, the first number represents the number of significantly affected transcripts.

Several relative expression values of Na^+^/K^+^-ATPase subunits (*at1a*, *nkai*) and also of plasma membrane Ca^2+^ ATPase (*at2b*) were significantly elevated in the comparisons EF and ED. Surprisingly, two DEGs coding for V-type ATPase (*vatb2* and *vatl*) were down-regulated in EF, but the relative expression value of additional 22 transcripts encoding for V-type proton ATPase were not significantly modified at the end of the exercise protocol. In ED, *vatb2* was also reduced, but the relative expression values of a transcript of a 16 kD proteolipid subunit (*vatl*) were elevated. A zinc transporter (*s39a4*) was elevated in EF, and also a ligand-gated ion channel with relatively high calcium permeability (*p2rx5* purinoceptor) was highly expressed and the relative expression value even increased. In ED, a transcript of the purinoceptor *p2rx1* was down-regulated, whereas *p2rx5* was up-regulated. Several transcripts of chloride intracellular channel protein 4 (*clic4*) were highly elevated in EF and ED. Relative expression values of a voltage-dependent anion-selective channel protein (*vdac*) were elevated from 988 to 2,624 in ED, but this elevation was not observed in EF. In ED, a voltage dependent Ca^2+^ channel (*ca2d2*) and a sodium channel (*scn1b*, *scn8a*) were significantly reduced in their relative expression value. As in EF, the highly expressed zinc transporter (*s39a4*) was significantly elevated in ED. On the other hand, the expression changes of several additional zinc transporters (*znt1*, *znt2*, *znt6*, *s39a5*, *s39aa*) expressed at low level (i.e. below 50 copies) were either elevated or reduced in their relative expression value in ED, without revealing a clear trend. For each of the sodium-hydrogen exchangers 5, 7, and 8 (*sl9a5*, *sl9a7*, *sl9a8*) more than 800 corrected reads were detected, but there was no difference between SC and EF. Several glucose transporter transcripts were affected in DED, but in contrast to EF one *gtr1* transcript was reduced in the relative expression level, and in particular *gtr5*, which was highly expressed and reduced in EF, was elevated in DED. In contrast to ED and to EF, several monocarboxylate transporter transcripts were reduced in DED. The increase in the relative expression values of voltage-dependent anion-selective channel protein (*vdac*) even exceeded the increase observed in ED. With respect to ion transport, the changes observed in DED mostly resembled the changes detected in ED.

### Intracellular signaling

A selection of DEGs related to intracellular signaling is presented in Table 5. Three DEGs of serine threonine-protein kinase (*sgk1*) were up to 5-fold elevated in EF and in ED. cAMP-dependent protein kinase type II-alpha regulatory subunit (*kap2*) was significantly elevated in the comparisons EF, ED, and DED, and *kap0* was elevated only in EF. In EF, 3 DEGs of cAMP-dependent transcription factors (*atf1*, *atf3*, *atf4*) were elevated, whereas in ED and in DED, solely *atf1* and *atf4* were elevated. The relative expression values of 5-AMP-activated protein kinase (AMPK) subunit gamma-2 (*aakg2*) were highly elevated in EF and ED, but the catalytic subunit (*aapk2*) was down-regulated in ED and in DED. Noteworthy, the relative expression values of Hif-1α (*hif-1a*) were several-fold elevated in EF and ED, but in DED only one out of three transcripts was elevated in the relative expression level. Transcripts of Von Willebrand factor (*vwa1*, *vwf*) were reduced in their relative expression value, but in EF the reduction by far exceeded the reductions seen in ED and DED. In contrast to EF, 5 independent transcripts of a 65 kDa serine threonine-protein phosphatase regulatory subunit (*2aaa*, *2aab*) were several-fold elevated in ED, and 4 in DED. MAP-kinase 1 (*m3k1*) was reduced in EF, in ED *m3k1* and *m3k3* were reduced. Several MAP-kinase transcripts were reduced in DED, but two out of three *m3k7* transcripts were elevated, while a third transcript was reduced in the relative expression level. In ED, muscarinic Ach receptor (*acm2*) was down-regulated, while beta-4c-adrenergic receptor (*adb4c*) was elevated in ED and DED.

**Table 5:** Selection of DEGs in EF, ED, and DED assigned to the term ‘intracellular signaling’, and the relative expression value of the respective genes detected in control groups SC and SD.

Intracellular signaling	SC	EF	ED	SD	DED
	relative expression value	fold change	fold change	relative expression value	fold change
**Serine Threonine-Protein Kinase**					
*sgk1 (5x)*, *sgk3*	25–180	↑ (4/6)	↑ (4/6)	80–1150	↑ (5/6)
**cAMP-dependent Protein Kinase**					
*kap0*, *kap1*	5–40	↑ (1/2) - ↓↓ (1/2)	-	5	↓ (1/2)
*kap2 (2x)*	25	↑ (1/2)	↑ (1/2)	15–30	↑
**cAMP-dependent Transcription Factor**					
*atf1*, *atf3*, *atf4*	20–405	↑	↑ (2/3)	35–600	↑ (2/3)
**cAMP-responsive Element Binding Protein**					
*creb5*	35	↑	-	40	↑
**5-AMP-activated Protein Kinase Subunit Gamma**					
*aakg1*, *aakg2 (2x)*	5–15	↑ (1/3) - ↑↑ (1/3)	↑↑ (1/3)	5–10	↑ (2/3) - ↓ (1/3)
**5-AMP-activated Protein Kinase Catalytic Subunit**					
*aapk1*, *aapk2*	70	-	↓ (1/2)	75–270	↓
**Hif-1α**					
*hif1a (3x)*	5–15	↑	↑	10	↑ (1/3)
**Von Willebrand Factor**					
*vwa1 (2x)*	40–50	↓ (1/2) - ↓↓ (1/2)	↓ (1/2)	55	↓ (1/2)
*vwf*	295	↓	-	-	-
**Peroxisomal Proliferator-activated Receptor α-Interacting Complex**					
*pr285*	3715	↓	↓↓	3125	↓↓
**Peroxisomal Proliferator-activated Receptor**					
*ppar (3x)*	140	↑ (1/3)	-	10–20	↓ (2/3)
**Serine Threonine-Protein Phosphatase Regulatory Subunit**					
*2aa (5x)*	15–60	-	↑	20–110	↑ (4/5)
**MAP-Kinase**					
*m3k1 (3x)*, *m3k3*	10–1205	↓ (1/4)	↓ (3/4)	350–845	↓ (3/4)
*m3k4*, *m3k5 (2x)*, *m3k6 (2x)*, *m3k7 (3x)*, *m3k11*	-	-	-	5–820	↑ (3/9) - ↓ (6/9)
**Acethylcholin Receptor**					
*acm (3x)*, *abd4c*	65–85	-	↑ (1/4) - ↓ (1/4)	20–110	↑ (2/4) - ↓ (2/4)

Arrows indicate increased (upwards) or decreased (downwards) expression value; a single arrow indicates up to 10-fold elevation/reduction, two arrows indicate more than 10-fold elevation/reduction. Numbers in brackets give the number of transcripts for the respective gene. If two numbers are listed, the first number represents the number of significantly affected transcripts.

### Extracellular matrix

Searching for the term ‘extracellular matrix’ within the GO domain *cellular component* revealed a remarkable number of transcripts that were significantly modified in EF (Table 6). Transcripts of disintegrins (*ats1*, *ats2*, *ats3*, *ats13*, and *ats 15*), matrix metalloproteinase (*mmp19* and *mmp23*), collagen transcripts (*co2a*, *co4a*, *co6a*), transcripts of fibronectin (*finc*), of laminin (*lama*), and also of decorin (*pgs2*) showed significantly higher relative expression values. Tight junction protein (*zo1*) increased several-fold in EF, ED, and in DED. The expression of zona pellucida genes (*zp1*, *zp2*, *zp3*) was terminated in EF and in ED, but one transcript was even elevated in DED. In ED, collagen transcripts (*co7a*, *co9a*), 2 laminins (*lam2*, *lama3*), lysyl oxidase (*loxl4*), and fibroblast growth factor receptor transcripts (*fgfr2* and *fgfr3*) were elevated. In contrast, in DED collagen and also laminin transcripts were either up- or down-regulated in their relative transcription level. A transforming growth factor beta receptor transcript *(tgbr*) was not affected in EF, but showed reduced relative expression values in ED and DED.

**Table 6:** Selection of DEGs in EF, ED, and DED assigned to the term ‘extracellular matrix’, and the relative expression value of the respective genes detected in control groups SC and SD.

Extracellular matrix	SC	EF	ED	SD	DED
	relative expression value	fold change	fold change	relative expression value	fold change
**Disintegrin**					
*ats1*, *ats2 (2x)*, *ats3*, *ats13*, *ats15*	5–605	↑ (5/6) - ↑↑ (1/6)	↑ (2/6) - ↓ (1/6)	-	-
*ats6*, *ats17*	5–45	-	↓ (1/2)	15–45	↓
**Matrix Metalloproteinase**					
*mmp2*, *mmp14*	-	-	-	420–7915	↓
*mmp19*, *mmp23*	10–40	↑	-	5	↑ (1/2)
**Collagen**					
*co2a*, *co4a (3x)*	50–1920	↑ (2/4) - ↑↑ (2/4)	-	-	-
*co5a*, *co6a (4x)*	75–195	↑ (2/5)	-	5–5935	↓
*co7a*, *co9a (2x)*	< 5–880	-	↑ (1/3) - ↑↑ (2/3)	5–460	↑ (2/3)
**Fibronectin**					
*finc (4x)*	40–305	↑ (3/4) - ↓ /1/4)	-	-	-
**Laminin**					
*lam2*, *lama2*, *lama3*, *lama5*	5	↑ (1/4)	↑ (1/4) - ↑↑ (1/4)	5–10	↑ (2/4)
*lama1*	-	-	-	130	↓
**Decorin**					
*pgs2*	175	↑	↓	7415	↓
**Tight Junction Protein**					
*zo1 (2x)*	45	↑ (1/2)	↑ (1/2)	35–45	↑
**Zona Pellucida**					
*zp1*, *zp2*, *zp3 (4x)*	40–200	↓↓ (1/6)—-∞ (5/6)	↓↓ (5/6)—-∞ (1/6)	20	↑ (1/4)
**Lysyl Oxidase**					
*loxl4*	5	-	↑↑	10	↑↑
**Fibroblast Growth Factor Receptor**					
*fgfr2 (3x)*, *fgfr3 (4x)*, *fgfr4*	5–50	-	↑ (3/8)	10–4625	↑ (6/8) - ↓ (2/8)
**Transforming Growth Factor Beta Receptor**					
*tgbr3*	1350	-	↓	1320	↓

Arrows indicate increased (upwards) or decreased (downwards) expression value; a single arrow indicates up to 10-fold elevation/reduction, two arrows indicate more than 10-fold elevation/reduction. Numbers in brackets give the number of transcripts for the respective gene. If two numbers are listed, the first number represents the number of significantly affected transcripts.

### Tissue homeostasis and repair

To identify DEGs coding for proteins connected to tissue homeostasis and repair, the GO domain *biological process* was searched for the terms ‘apoptosis’, ‘inflammatory’, and ‘immune response’. Transcripts of angiopoietin1 short (*angp1*), 5 rho guanine nucleotide exchange factor transcripts (*arhg2*, *arhgb*, *arhgc*, *arhgf*, *arhgp*), semaphorin (*sem3f*, *sem4e*, *sem5a*), and prostaglandin synthase 2 (*pgh2*) showed significantly elevated relative expression values in EF (Table 7). In contrast to EF, in ED semaphorin transcripts (*sem3d*, *sem3f*) were reduced in their relative expression value, and the relative expression values of *sem4b* and *sem4g* were either reduced or elevated. Even more semaphorin transcripts were affected in DED, and as observed in ED, the relative expression level was either reduced or elevated. 6 myosin-xviiia transcripts (*my18a*) were elevated in EF, but not in ED, and in DED two were even reduced. Five out of seven transcripts of cytochrome p450 (*cp26a*, *cp2dq*, *cp2k1*, *cp2k1*, *cp2k3*, *cp2m1*) and one transcript of NADPH-dependent cytochrome p450 reductase (*ncpr*) were several-fold elevated in EF, while in ED several cytochrome p450 transcripts were reduced in their relative expression value. In DED, the relative expression level was either reduced or elevated.

**Table 7:** Selection of DEGs in EF, ED, and DED connected to the terms ‘apoptosis; inflammatory; and immune response’, and the relative expression value of the respective genes detected in control groups SC and SD.

Tissue homeostasis and repair	SC	EF	ED	SD	DED
	relative expression value	fold change	fold change	relative expression value	fold change
**Cytochrome p450**					
*cp1b1*, *cp26a*, *cp26b (2x)*, *cp2u1*	90–130	↑ (1/5) - ↓ (1/5) - ↓↓ (1/5)	↓ (2/5) - ↓↓ (1/5)	25–440	↓ (2/5) - ↓↓ (1/5)
*cp2k1 (2x)*, *cp2k3*, *cp2dq*, *cp2m1*	< 5–125	↑ (3/5) - ↑↑ (1/5) - ∞ (1/5)	↑ (1/5)—-∞ (1/5)	< 5–100	↑ (2/5) - ↑↑ (3/5)
**Angiopoietin short**					
*angp (4x)*	5–15	↑ (3/4)	-	10	↓ (1/4)
**Rho Guanine Nucleotide Exchange Factor**					
*arg33*, *argh37*, *arhg1*, *arhg2*, *arhg6*, *arhg8 (2x)*, *arhg9*, *arhgb*, *arhgc (2x)*, *arhgf*, *arhgi*, *arhgp*, *arhgq*	15–40	↑ (5/15)	↑ (2/15)	< 5–660	↑ (4/15) - ↑↑ (1/15) - ↓ (7/15)
**Myosin-18**					
*my18a (6x)*	5–55	↑ (3/6) - ↑↑ (3/6)	-	10–30	↓ (2/6)
**Semaphorin**					
*sem3b*, *sem3c*, *sem3d (2x)*, *sem3f (2x)*, *sem5a*, *sem6d*	< 5–840	↑ (1/8) - ↑↑ (1/8)	↓ (2/8)	65–540	↑ (1/8) - ↓ (6/8)
*sem4b (2x)*, *sem4e*, *sem4g*,	15–420	-	↓ (1/4) - ↑ (1/4) - ↑↑ (1/4)	15–655	↑ (2/4) - ↑↑ (1/4) - ↓ (1/4)
**Prostaglandin Synthase**					
*pgh1*, *pgh2*	70–780	↑ (1/2)	↓ (1/2)	50	↓ (1/2)
**nlrc3**					
*nlrc3 (11x)*	15–60	↓ (1/11) - ↓↓ (1/11)	↑	5–80	↑ (9/11) - ↓ (2/11)
**NADPH-dependent Cytochrome p450 Reductase**					
*ncpr*	5	↑	↑	5	↑
**Death-associated Protein Kinase**					
*dapk1 (2x)*, *dapk2*, *dapk3*	380	-	↓ (1/4)	20–1785	↑ (1/4) - ↓ (3/4)
**Immunity related GTPase Imap Family Member 7**					
*gima7 (5x)*	5–465	-∞ (1/5)	↓ (3/5) - ↓↓ (2/5)	35–485	↓ (3/5)
**Rho GDP-dissociation Inhibitor**					
*gdir1 (4x)*, *gdir2 (2x)*	2565	-	↓ (1/6)	20–2930	↑ (1/6) - ↓ (5/6)
**Receptor-interacting Serine-Threonine Protein Kinase**					
*ripk2*, *ripk4*	405	-	↓ (1/2)	545–900	↑ (1/2) - ↓ (1/2)
**Interferon-induced 35 kDa Protein**					
*in35*	910	-	↓	580	↓
**Interleukin-23 Receptor**					
*il23r*	120	-	↑	185	↑
**Proteasome Subunit Beta Type**					
*psb2*, *psb4*, *psb5*	-	-	-	45–275	↑
*psb7 (2x)*	15–270	-	↑	20–300	↑
**Nociception Receptor**					
*oprx (2x)*	< 5–150	-	↑ (1/2) - ↑↑ (1/2)	< 5–165	↑ (1/2) - ↑↑ (1/2)
**ATP-binding Cassette ATPases**					
*abcf1 (3x)*, *abcf2 (7x)*, *abcf3*	< 5–125	↑ (2/11)	↑ (8/11) - ↑↑ (1/11)	10–685	↑ (9/11)

Arrows indicate increased (upwards) or decreased (downwards) expression value; a single arrow indicates up to 10-fold elevation/reduction, two arrows indicate more than 10-fold elevation/reduction. Numbers in brackets give the number of transcripts for the respective gene. If two numbers are listed, the first number represents the number of significantly affected transcripts.

In ED and in DED, several transcripts of death-associated protein kinase (*dapk*), of immunity related gtpase imap family member 7 (*gima7*), and of rho gdp-dissociation inhibitor 2 (*gdir2*) were reduced. 15 Rho guanine nucleotide exchange factor transcripts were either up or down-regulated in DED, while only two of these transcripts were elevated in ED. In contrast to EF, receptor-interacting serine-threonine protein kinase 2 (*ripk2*) and also the relative expression value of interferon-induced 35kda protein (*in35*) were reduced in ED and DED, while interleukin-23 receptor (*il23r*) was elevated. Compared with EF, in ED and in DED the relative expression value of several transcripts of protein *nlrc3* and also of proteasome subunit beta type 7 (*psb7*) were elevated following exercise. In contrast to EF, where only 2 members of the atp-binding cassette atpases were elevated after exercise, in both, ED and DED, 9 transcripts of these atpases (*abcf1*, *abcf2*, *abcf3*) were significantly elevated.

## Discussion

Recordings from tagged silver eels during their spawning migration to the Sargasso Sea revealed that eels swim about 12 to13 h a day at lower depth of about 200 m, and 8 to 9 h under elevated pressure at greater depth [5, 16]. In our experiments, eels had to swim for 8 h under elevated pressure prior to tissue sampling, which resembled the time eels swim under elevated pressure in nature, although there the hydrostatic pressure is significantly higher, which for our experiments was not possible for technical reasons. Due to the design of our study, changes in transcription could be related to swimming alone, or to swimming under elevated pressure. Eels have repeatedly been analyzed in a swim tunnel under atmospheric pressure [4, 33, 46], illustrating the effect of swimming on energy metabolism. The data showed that European eels are capable of swimming for several months in a swim tunnel with remarkable low cost of transport [47–49], and fat, protein, and carbohydrate contributed to energy metabolism [49]. Swimming under constant atmospheric pressure will not affect the buoyancy status, therefore activation of gas gland cells and stimulation of gas secretion is not required. Actually, activation of gas secretion would even be counterproductive, because it will create a status of positive buoyance, which will result in an elevated energy expenditure to prevent surfacing [20]. Therefore, we restricted our study to swimming under elevated pressure, which is connected to changes in buoyancy.

Although the total number of DEGs was not too different between exercised silver eels with functional and with damaged swimbladder, in ED almost twice as many DEGs showed an elevated transcription, while in EF, the number of DEGs with an elevated or lowered transcription was more balanced. Comparing silver eels with damaged swimbladder (SD) with exercised silver eels with heavily infected and damaged swimbladder revealed a slightly higher number of elevated than lowered DEGs, but the total number was more than twice as high than in ED. Analyzing the effect of silvering on transcriptional activity in infected and non-infected eels also revealed a much higher number of affected transcripts in infected eels [50]. This could indicate that the infection of the swimbladder reduces the stress resistance, so that hydrostatic pressure changes encountered in the swim tunnel provoke especially pronounced changes in transcription. A previous study also revealed that transcriptional activity in yellow eels was much more responsive to the infection than in silver eels, indicating different sensitivity during different developmental stages [42].

The difference in the response of the two exercise groups was obvious when looking at the results for the GO analysis with respect to *biological process*. None of the significantly affected functions occurred simultaneously in EF and in ED, and only two processes in EF and in DED, emphasizing that compared to eels with a functional swimbladder, eels with a damaged swimbladder showed large scale differences in the transcriptional changes in swimbladder tissue following exercise under elevated hydrostatic pressure. This finding was supported by the Venn diagram, revealing that damage of the swimbladder due to the infection with *Anguillicola crassus* largely influences the number of DEGs. This was also supported by looking at DEGs that, based on our current knowledge, are crucial for swimbladder function.

According to Boyle’s law, an increase in hydrostatic pressure causes a decrease in swimbladder volume, so that gas secretion should be stimulated in order to compensate the volume decrease. Gas secretion is stimulated by increasing acid secretion via lactic acid production in glycolysis and via CO_2_ production in the pentose phosphate shunt (PPS) [7, 51–54]. Swimming for 8 h under elevated hydrostatic pressure indeed resulted in a significant elevation of transcription of genes involved in energy metabolism, in particular in genes coding for glycolytic enzymes, irrespective whether the swimbladder was fully functional, or had previously been infected by the nematode *Anguillicola crassus* and was therefore seriously damaged, so that it could no longer serve as a buoyancy structure. The elevated transcription of glycolytic enzymes, in particular of lactate dehydrogenase, suggested that glycolysis was stimulated in order to acidify the blood and to stimulate gas secretion into the swimbladder by switching on the Root effect [9, 11]. This conclusion was supported by an elevation of *gtr1* transcripts, as an elevation of glucose transporter 1 protein may facilitate glucose uptake by gas gland cells, fueling the glycolytic pathway. This was also supported by the enhanced transcription of zinc transporter transcripts (*s39a4*) and of carbonic anhydrase (*cahz*), a zinc containing enzyme playing a crucial role in CO_2_ exchange between gas gland cells and the blood, and in CO_2_ secretion [55, 56].

Acid secretion is achieved via V-type ATPase activity and via NHE (Na^+^/H^+^ exchanger) proteins [55, 57], but transcription of these membrane proteins was not enhanced. On the other hand, the relative expression values of V-type ATPase transcripts and of transcripts of NHE proteins 5, 7, and 9 were already high in SC animals, probably providing sufficient capacity for acid release even with elevated glycolytic activity. Acid release via NHE proteins would require enhanced sodium excretion to compensate for the sodium entry into the gas gland cells, and the relative expression value of Na^+^/K^+^-ATPase subunit transcripts was indeed elevated in EF.

A theoretical analysis of the capacity of the swimbladder for acid secretion revealed that full compensation of the changes in swimbladder volume due to the pressure changes encountered during the daily migrations would not be possible over the time of several months [20]. However, the previously described histological changes [22] and the transcriptional changes detected in our study clearly show that the swimbladder is active in migrating eels. Although keeping a constant volume of the swimbladder during the daily migrations is probably impossible, gas secretion obviously does occur. The flexible-walled swimbladder is not totally gas tight, and therefore the large pressure changes encountered during the daily migration must result in a loss of gas to the surrounding tissues, in particular because the swimbladder gas mainly consists of oxygen [19], while the main gas in water is nitrogen. In addition, in water gas partial pressure does not increase with increasing depth. Accordingly, in order to avoid a progressive decrease in swimbladder volume, some gas secretion must occur. Therefore, with elevated hydrostatic pressure gas gland cell acid secretion and subsequent gas secretion was stimulated. Even though a full compensation appears not possible, our results clearly show that the swimbladder does contribute to buoyancy regulation in migrating eels.

These changes in the expression of glycolytic enzyme transcripts were more or less similarly detected in EF and ED, and similar changes were also detected when comparing heavily infected non-exercised silver eels with exercised silver with a damaged swimbladder (DED). This was quite unexpected as heavily damaged swimbladders show an almost gasless lumen and therefore glycolysis is obviously not necessary to facilitate gas deposition into the lumen. However, it may be that the enhanced transcription of glycolytic genes in exercised eels with a damaged swimbladder was at least partly stimulated by an elevated thickness of the tissue, which decreases oxygen supply to the tissue due to increased diffusion distances. A significant difference between EF and ED was the decrease in a carbonic anhydrase transcript (*cah12*) and in particular the elevated transcription of *hxk2* in ED, and this was also seen in DED. While hexokinase 1 normally phosphorylates glucose on cell entry, in tumor cells hexokinase 2 has been shown to be crucial for the Warburg effect, i.e. a shift to aerobic glycolysis for ATP production, irrespective of the availability of oxygen [58]. Noteworthy is the concomitant elevation of *vdac* transcripts in ED and DED, as this voltage dependent ion channel has been shown to bind hexokinase 2 to the outer mitochondrial membrane [59]. In contrast to EF, in ED and even more so in DED, mitochondrial NADH-dehydrogenase was elevated at the transcription level, which also supported the conclusion that the modification of metabolism in ED was not directed towards an increased acid production, but mainly the result of thickened tissue and prolonged diffusion distances. This was further supported by the observation that compared with EF, in ED and in DED, changes in carbonic anhydrase transcription were inconsistent. Carbonic anhydrase facilitates CO_2_ diffusion and significantly contributes to blood acidification and initiation of the Root effect, which is not essential if the anaerobic metablism is related to a thickened tissue in exercised eels with a damaged swimbladder.

The conclusion that swimming under elevated hydrostatic pressure induced adjustments in gas gland cell metabolism was supported by the observation that the relative expression value of AMPK transcripts was significantly elevated. AMPK is a critical energy sensor, but may also be a glucose sensor switching on catalytic pathways [60, 61] and thus glucose metabolism. In addition, cAMP-dependent protein kinases and cAMP-dependent transcription factors showed an increased transcription, which are also typically involved in metabolic adjustments, signal transduction, and stress responses [62]. cAMP and protein kinase A (PKA) have previously been shown to be involved in the regulation of acid secretion in eel gas gland cells [63]. The high relative expression value of guanine nucleotide exchange factor transcripts detected with an elevated level of transcription suggested that extracellular signals elicited the modification of metabolic activity, as the Rho GTPase family of proteins is involved in signal transduction [64].

Unexpected was the elevation of hif-1α and the decrease of von Willebrand factor transcripts in EF and ED. Hif-proteins are activated under hypoxic conditions [65], and the swimbladder containing oxygen as the main gas certainly will not experience hypoxia, especially not under hyperbaric pressure. In addition to hypoxic signaling, Hif proteins are involved in developmental processes and organ formation in zebrafish [66], and therefore it appears possible that the elevated transcription was related to organ reconstruction, and not to hypoxic signaling.

Prior to starting the spawning migration, eels traverse the process of silvering, and this is connected to a significant reconstruction of the extrcellular matrix of the swimbladder, most likely to improve its gas impermeability [21–23, 50]. This was in line with the significant elevation of several extracellular matrix related transcripts in particular in EF. Remarkable was the increase in *my18a* transcription, which was not observed in ED. Myosin18a comprizes binding sites for actin and also for the Golgi apparatus (GOLPH3) and therefore is important for vesicle formation and transport [67]. The reconstruction of the extracellular matrix requires the production of secretory proteins and therefore the activity of the Golgi apparatus, and in EF a large number of extracellular matrix related transcripts was elevated in the relative expression value. These changes not only included various components of the extracellular matrix, but also extended to matrix metalloproteinases, indicating that a reconstruction of the extracellular matrix occurred, most likely to improve gas impermeability. Tight junction proteins were also affected, suggesting that the connection between the epithelial gas gland cells was tightened. In ED and in DED, the effect on extracellular matrix transcripts was much less pronounced, indicating that the reconstruction of the extracellular matrix in eels with damaged swimbladder was not as extensive as in eels with a functional swimbladder.

In EF, angiopoietin transcripts were elevated, suggesting that blood vessel formation was stimulated. In addition, the relative expression value of a semaphorin transcript increased. While semaphorins have originally been described as factors involved in axon guidance, recent evidence revealed that they may have anti-inflammatory function and may stimulate endothelial cells supporting angiogenesis [68]. An increase in the length of rete mirabile capillaries has been detected during silvering [21, 23], thus the increased transcription of angiopoiesis related genes is in line with an improvement of swimbladder blood supply and an improved countercurrent system.

In EF, the relative expression values of several cytochrome p450 transcripts was significantly elevated, while in ED the relative expression values were mostly reduced. Cytochrome p450 is a unique hemeprotein, evolved to protect organisms against toxic compounds [69]. These monooxygenase enzymes that may also contribute to steroid metabolism, are related to stress and may function as intracellular sensor for gases like O_2_, CO, and NO, which may explain the elevated transcription in gas gland cells [70, 71]. They could even sequester free radicals, which may be formed at high oxygen partial pressures [70].

Overall, in ED by far more transcripts related to tissue homeostasis, apoptosis, and immune response have been detected, and a large number of these DEGs were not affected or even modified in the opposite direction in EF. Thus, these expression changes revealed that in ED an immune response was elicited, which is in line with a previous heavy infection with the nematode *Anguillicola crassus*, damaging the swimbladder as observed in our studies [27, 28, 72]. This conclusion is also in line with the Venn diagram, showing that the swimbladder damage is a main factor determining the number of DEGs. While the relative expression value of mitogen-activated protein kinase (*m3k1*, *m3k3*) was reduced in ED and DED, several transcripts of serine threonine-protein phosphatase and -kinase were highly expressed and significantly elevated, indicating that the MAPK pathway is of particular importance in exercised eels with a damaged swimbladder. In EF, only one single *m3k* transcript of this pathway was significantly reduced in the expression level. Serine threonine-protein kinases are involved in the regulation of a wide variety of ion channels, membrane transporters, cellular enzymes, transcription factors, but also cell growth, proliferation, survival, and apoptosis [73, 74]. In particular, *sgk1* (3 transcripts affected) plays an important role in cellular stress responses, in electrolyte balance, and inflammation [73]. The observed transcriptional changes in ED therefore clearly demonstrate a defense response in gas gland tissue, as already observed when the influence of an *Anguillicola crassus* infection on the silvering process was assessed [50].

An interesting though unexpected observation in our experiments was the significant effect of the exercise protocol under elevated hydrostatic pressure on transcription of genes related to the circadian clock. The recently described diurnal vertical migrations [5, 13–16] may be connected to the activity of clock genes, and in zebrafish it has been shown that even in peripheral cells and in cell lines the clock is established and entrainable [75, 76]. The diurnal vertical migrations directly affect swimbladder pressure and volume, and therefore it is conceivable that circadian clock genes are active in gas gland cells. Based on a chemical clue, for example, rhythmic activity of gas gland cell metabolism and acid secretion could be elicited and coordinated with the depth changes encountered during the vertical migration. Although a full compensation and a constant swimbladder volume does not appear possible, some contribution to buoyancy regulation would reduce the overall energy expenditure required for these daily excursions [20].

In conclusion, our results show that swimming under elevated hydrostatic pressure elicited significant transcriptional changes in swimbladder gas gland cells. These changes were in line with a stimulation of glucose metabolism, resulting in an elevated acid production and secretion in order to switch on the Root effect and support gas secretion into the swimbladder. Although gas secretion does not appear to be sufficient for full compensation of the volume changes induced by the elevated hydrostatic pressure, it may compensate for diffusional gas loss out of the swimbladder. The results therefore support the conclusion that the swimbladder does contribute to buoyancy regulation in migrating eels, although a status of neutral buoyancy may not be achieved throughout the diurnal vertical migrations. The results also support the notion obtained from a previous study evaluating the modifications of the swimbladder related to the process of silvering [42, 50], that an infection of the swimbladder with the nematode *Anguillicola crassus* and the resulting damage significantly impairs swimbladder function.

## Supporting information

S1 Table (XLSX)

S2 Table (XLSX)

S3 Table (XLSX)
